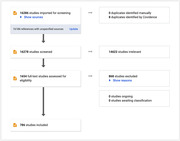# Measurements of decision‐making ability in middle to late adulthood: a multi domain scoping review from the ARMCADA study

**DOI:** 10.1002/alz.093481

**Published:** 2025-01-03

**Authors:** Emily Ho, Berivan Ece, Zahra Hosseinian, Molly A Mather, Elizabeth Dworak, Miriam Novack, Tatiana Karpouzian‐Rogers, Sarah Pila, Sandra Weintraub, Richard C. Gershon

**Affiliations:** ^1^ Northwestern University Feinberg School of Medicine, Chicago, IL USA; ^2^ Northwestern University, Chicago, IL USA; ^3^ Northwestern, Chicago, IL USA

## Abstract

**Background:**

Deficits in decision‐making (DM) can lead to adverse outcomes across multiple domains such as financial management and medical care. By hindering such DM abilities, cognitive impairment (CI) often affects quality of life. Routine screening for CI, however, does not include systematic and comprehensive assessment of DM ability. While there are many measures of DM ability, there is considerable heterogeneity in what constructs are measured and the populations in which they have been validated. This scoping review assessed what decision‐making measures were present in the literature and their validation evidence, across the domains of financial, functional outcomes, health care, end‐of‐life, and affective.

**Method:**

We adhered to established scoping review methodology and included keywords pertaining to measurement, technology, decision‐making, and age across a variety of databases. A total of 16278 articles were identified, and of those, 1654 passed title and abstract screening, and were deemed eligible for full‐text review. Seven‐hundred eighty six studies were included for consideration of full‐text extraction; currently, 581 were identified for full‐text extraction.

**Result:**

Preliminary findings suggest that across domains, there were more measures for functional outcomes (25%), health‐care decision‐making (19%), followed by financial decision‐making (15%), affective (15%), and end‐of‐life (3%), though there were many measures rated as encompassing more than one category. Measures are overwhelmingly in‐person (69%), while 16% could be administered remotely, although many of them were computer‐based tasks. Common decision‐making tasks included tests of risk (e.g., gambling tasks), semi‐structured interviews of assessment, and brief cognitive screenings. Such measures were presented with a variety of clinical populations.

**Conclusion:**

Many measures of decision‐making ability identified were used in laboratory or research settings, or measured one construct. Future work will investigate adaptation, validation, and development of existing decision‐making measures, for early detection of preclinical dementia.